# Altered chemosensitivity to CO_2_ during exercise

**DOI:** 10.14814/phy2.14882

**Published:** 2021-06-10

**Authors:** Stanley M. Yamashiro, Takahide Kato, Takaaki Matsumoto

**Affiliations:** ^1^ Biomedical Engineering Department University of Southern California Los Angeles CA USA; ^2^ Department of General Education National Institute of Technology Toyota College Toyota Japan; ^3^ School of Health and Sport Sciences Chukyo University Toyota Japan

**Keywords:** chemosensitivity, CO2 inhalation, exercise

## Abstract

The effect of exercise on chemosensitivity to carbon dioxide (CO_2_) has been controversial. Most studies have been based on rebreathing to alter inspired CO_2_ which is poorly tolerated in exercise. Instead, inhaling a fixed 3% CO_2_ from rest to moderate exercise was found to be well tolerated by seven normal subjects enabling CO_2_ chemosensitivity to be studied with minimal negative reaction. Results showed that chemosensitivity to CO_2_ following 5–6 min of stimulation was significantly enhanced during mild exercise (*p *< 0.01). This motivated exploring how much of the dynamic ventilatory response to mild exercise breathing air could be predicted by a model with central and peripheral chemosensitivity. Chemoreceptor stimulation combined with hypercapnia has been associated with long‐term facilitation of ventilation (LTF). 3% CO_2_ inhalation during moderate exercise led to ventilation augmentation consistent with LTF following 6 min of exercise in seven normal human subjects (*p *< 0.01). Increased ventilation could not be attributed to hypercapnia or metabolic changes. Moderate exercise breathing air resulted in significantly less augmentation. In conclusion, both peripheral and central chemosensitivity to CO_2_ increased in exercise with the peripheral chemoreceptors playing a dominant role. This separation of central and peripheral contributions was not previously reported. This chemoreceptor stimulation can lead to augmented ventilation consistent with LTF.

## Graphical abstract

Chemosensity to CO_2_ increases in exercise and promotes long term facilitation of ventilation.

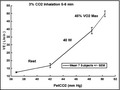

## 1 INTRODUCTION

Augmentation of chemosensitivity during mild exercise in normal man was reported by Weil et al. (1972) using variable inspired carbon dioxide (CO_2_). Both hypoxia and hypercapnia were studied so the peripheral chemoreceptors were felt to be involved, but a contribution by central chemoreceptors could not be ruled out. Miyamura et al. (1976) tried rebreathing of CO_2_ up to 10% inspired during exercise and concluded that CO_2_ depressed ventilation during exercise. More recently, Duffin et al. (1980) concluded no change in CO_2_ sensitivity during light exercise also based on rebreathing. The method of administration of CO_2_ may have been a contributing factor in explaining these divergent results. Inspired CO_2_ of 7.5% or more is known to stimulate subject arousal and anxiety (Savulich et al., 2019). An inspired level of 3% was felt to avoid such negative reactions and was used in the present study to test this hypothesis. The duration of CO_2_ inhalations is another important factor and 5–6 min was felt long enough to observe both peripheral and central chemoreceptor responses and short enough to minimize subject discomfort. The relative roles played by central and peripheral chemoreceptors in any augmentation has also not been previously determined. Five to six minutes of transient response data should be sufficient to separately estimate central and peripheral contributions using model fitting (Bellville et al., 1979). Chemoreceptor gain is known to increase in hypoxia. The response to intermittent hypoxia when combined with hypercapnia in humans has led to long‐term ventilatory facilitation (LTF) (Griffin et al., 2012; Mitchell & Johnson, 2003; Wadhwa et al., 2008). If peripheral chemoreceptor stimulation can be significantly enhanced in exercise as discussed above, combined CO_2_ inhalation and exercise may also lead to LTF. LTF could be indicated by a ventilation increase following 5–6 min of exercise during hypercapnia. Whether this occurs is a question addressed in this study. Moderate exercise (45% maximum MRO_2_) was used to avoid anaerobic effects. Prior studies on LTF have primarily focused on intermittent hypoxic chemoreceptor stimulation using longer total durations. Use of higher levels of ventilatory stimulation in combined exercise and CO_2_ inhalation may help overcome this possible limitation. A hypercapnic background to intermittent hypoxia has been reported to be effective in producing LTF in humans using eight hypoxic episodes each 4 min long (Harris et al., 2006). Thus, whether 5–6 min of transiently applied combined moderate exercise and 3% inhaled CO_2_ does satisfy the intermittency and threshold requirements for LTF is the final addressed question.

## 2 MATERIALS AND METHODS

The experimental methods have been previously described (Kato et al., 2021). Ventilation responses were not the focus of previous use of the collected data and was the main topic in the present study. The current study was also limited to light and moderate exercise levels. An abbreviated summary is listed below.

### 2.1 Subjects

Seven healthy, active males (age 21.7 ± 0.5 years; height 171.6 ± 7.4 cm; body mass 64.5 ± 4.7 kg; VO_2max_ 44.1 ± 6.4 ml/kg/min; mean ± SD) with no history of cardiorespiratory diseases volunteered to participate in the present study. Informed consent was obtained from each subject after a full explanation of the experimental procedure as well as its risks was provided. The experimental protocol was approved by the Human Subjects Committee at the Chukyo University Graduate School of Health Sciences.

### 2.2 Maximal exercise test

Each subject performed an incremental exhaustive cycle exercise. Exercise test was conducted using an electrically braked cycle ergometer (AEROBIKE75XL; Combi Wellness); the workload was set at 40 watts (W) at the beginning of the test and increased by 20 W every minute until exhaustion. Subjects were encouraged to maintain a pedaling rate of 70 revolutions per minute (rpm). During the experiment, Oxygen uptake (V̇O_2_) was continuously analyzed using a breath‐by‐breath (BB) gas collection system and analyzed every 30 s using an automatic gas analyzer (RM300, MG360; Minato Medical Science). This system used a hot wire flowmeter to measure airflow and an infrared CO_2_ analyzer to measure breath CO_2_. Heart rate (HR) was also recorded every 30 s using a heart rate monitor (Life Scope B; Nihon Kohden). For assessment of VO_2max_, two of the following three criteria were satisfied: (1) identification of a plateau in VO_2_ with an increase in workload (≤150 ml increase), (2) HR ±10% of age‐predicted maximum (220 − age), and (3) RER ≥1.10.

### 2.3 Main experimental protocol

After 5 min rest session at sitting position on the cycle ergometer, subjects performed baseline cycling at 40W for 6 min. Continuously, subjects carried out the constant work‐rate exercise (CWE) at 45% VO_2 max_ intensity for 6 min using the same cycle ergometer as in the maximal exercise test. The pedaling rate of both baseline cycling and CWE sessions were 70 rpm. Each subject performed CWE tests on two occasions in normal barometric pressure, under the following conditions: (1) breathing ambient air (Air), (2) breathing enriched CO_2_ gas (CO_2_ 3.03 ± 0.06%; O_2_ 20.99 ± 0.03%; balance N_2_) (3% CO_2_). The subjects were blinded to the inhaled gas composition. The interval between each exercise test was at least a day. On the day before the exercise test, subjects were advised to avoid strenuous exercise, alcohol, caffeine, smoking, and to fast after dinner.

### 2.4 Measurement of respiratory responses

VO_2_, CO_2_ output (VCO_2_), minute ventilation (VE), end‐tidal partial pressure of O_2_ and CO_2_ (PetO_2_ and PetCO_2_, respectively), and tidal volume (V_T_) during steady state exercise were recorded using an automatic gas analyzer in the BB system. These data were averaged for every 30 s and output. PetCO_2_ during exercise or hypercapnia overestimates arterial partial pressure of CO_2_ (PaCO_2_), thus PaCO_2_ was estimated using the formula of Jones et al. (1979).

$${\text{Estimated PaCO}}_{2}=5.5+0.9*{\text{PetCO}}_{2}‐0.0021*V_{T}.$$

Data of these respiratory chemoreception factors were based on the mean values of the 5 min air rest period, last 30 s of resting and 40 W CO_2_ inhalation sessions and 4.5 min of CWE at 45% VO_2 max_ CO_2_ inhalation. Forty W exercise data with air breathing were analyzed using 6 min of data sampled every 30 s. The baseline VE and PetCO_2_ values used were the mean values of the 5 min air rest period.

### 2.5 Statistical analyses

For statistical comparisons of difference in the CO_2_ chemosensitivity, paired *t* tests were used. The statistical package (PASW statistics 25; SPSS) was used for statistical analysis. *p* < 0.05 were considered significant. We also calculated the effect size (*d*) with the following formula.

$$\text{Effect size}\;\left(d\right)=\frac{A_{\text{mean}}‐B_{\text{mean}}}{\sqrt{ASD^{2}+BSD^{2}/2}}.$$

## 3 RESULTS

**FIGURE 1:**
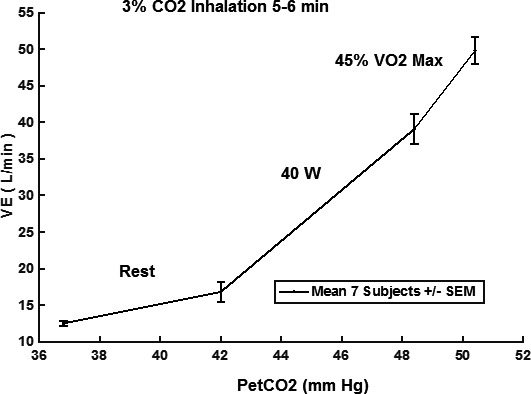
CO_2_ responses from rest to moderate exercise. VE, minute ventilation; P_etCO2_, end‐tidal partial pressure of CO_2_. Response slopes: rest 0.83, 40W 3.48, 45%VO_2max_ 5.35 (L/min)/mmHg

**FIGURE 2:**
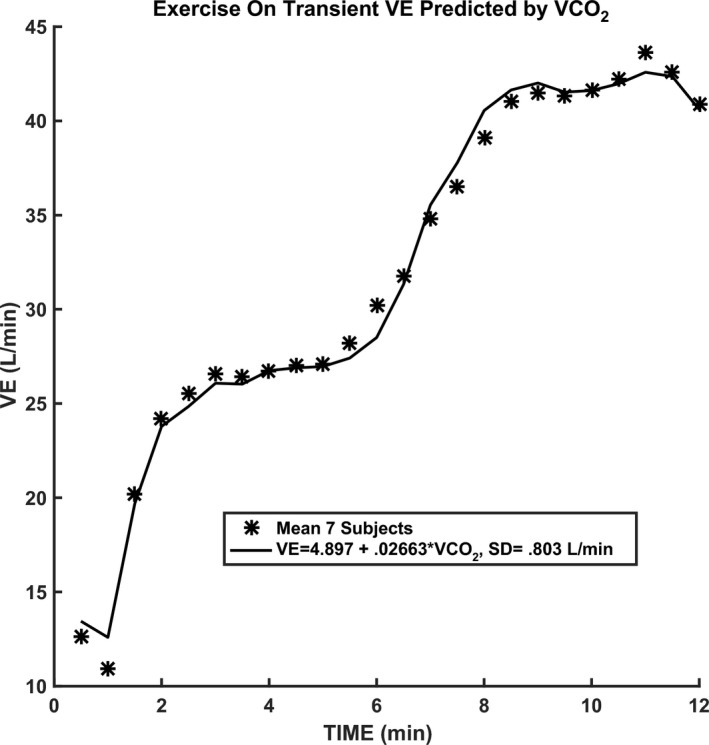
Correlation of ventilation with CO_2_ production rate in time. VE, minute ventilation; VCO_2_, minute carbon dioxide output

**FIGURE 3:**
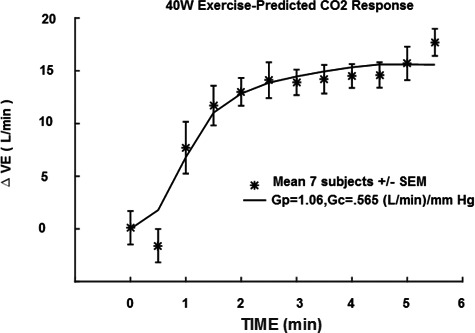
Central and peripheral model prediction compared to measured ventilation. VE, minute ventilation; *G*_p_, peripheral gain; *G*_c_, central gain

In Figure 1, the CO_2_ responses to 5–6 min of 3% CO_2_ are compared. Note that a significant increase in CO_2_ sensitivity from 0.83 to 3.48 (L/min)/mmHg was observed in the mean response for seven subjects for mild exercise. The change in sensitivity from mild to moderate exercise was less pronounced. In Table 1, a paired comparison of individual subject responses is shown. A statistically significant change (*p* < 0.01) was indicated. This was a primary goal of this study which then supported the hypothesized improved CO_2_ testing made possible with 3% inhalation and shortened CO_2_ exposure time. The question then shifted to the significance of increased sensitivity. In a proportional control system increased loop gain is expected to decrease control error (Khoo, 2001). Loop gain is an important determinant of respiratory control stability and is primarily determined by peripheral chemoreceptor sensitivity. In the present case, PaCO_2_ should be better controlled. One manifestation of overall control is the correlation of VE to metabolic production rate of VCO_2_. This is well established in the steady state. Figure 2 shows how measured VE can actually be closely predicted in time from this overall correlation, at least to a resolution of 30 s (VE and VCO_2_ sampling interval). In Figure 3, the model of Bellville et al. (1979) with central and peripheral chemoreceptor first order dynamics (see Appendix A) was used to fit ventilation responses to the measured PetCO_2_. Two parameters were estimated from least squares fitting using Matlab fminsearch. The estimated central gain (*G*_c_) was 0.565 and peripheral gain (*G*_p_) was 1.06 both in units of (L/min)/mmHg. The sum *G*_c_ + *G*_p_ = 1.6 can be compared to the resting total sensitivity of 0.83 estimated in Figure 1.

**TABLE 1:** Paired comparison of subject CO_2_ sensitivities in (l/min)/mmHg

Subject	Rest	40W	∆ (40W‐Rest)
1	0.12	2.26	2.14
2	1.95	3.33	1.38
3	1.54	4.68	3.14
4	0.61	4.72	4.11
5	0.71	3.79	3.08
6	1.70	2.35	0.65
7	0.90	6.41	5.51
Mean	1.08	3.93*	2.86
SD	0.67	1.47	1.65

40W, baseline cycling at 40W.

* *p* < 0.001, rest vs. 40W. *t*_(6)_ = −4.588, *p* = 0.004, *d* = 2.495.

**FIGURE 4:**
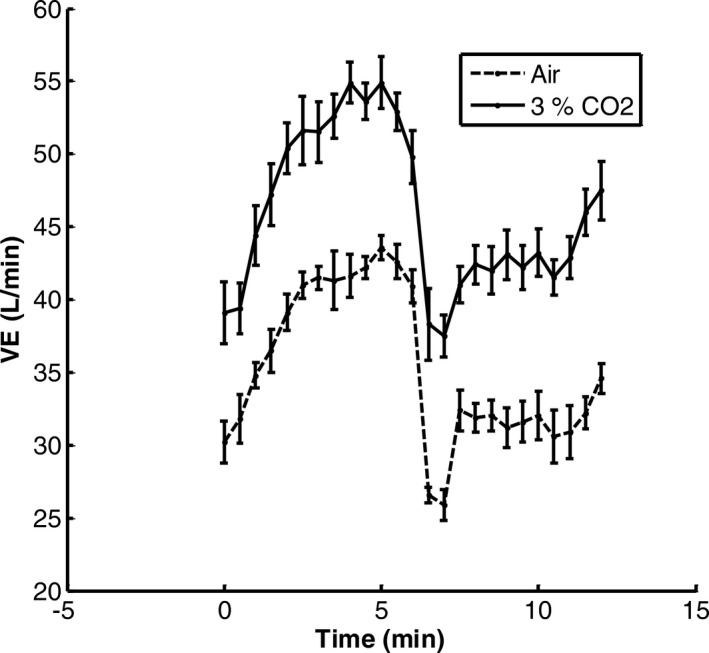
Averaged ventilation response in seven subjects to 45% VO_2max_ and 3% CO_2_ inhalation. Values are means ± SEM. Baseline was 40 W exercise and 3% CO_2_ inhalation. Averaged responses to 45% Maximum VO_2_ breathing air‐dashed line curve

The averaged transient response to 45% Max MRO_2_ while inhaling 3% CO_2_ is shown in Figure 4. Note that 40W exercise with 3% inhaled CO_2_ preceded and followed this moderate exercise episode. The main new observation is the increase in ventilation following recovery. Figure 4 also shows the transient response to the same exercise level while inhaling air. An increase in ventilation is still seen but at a smaller level. Table 2 compares ventilation measured over 30 s before 45% Maximum VO_2_ exercise and at the end of the recovery period of 40 W exercise. By paired comparison in seven subjects the mean change of 8.4 L/min was measured. Table 2 compares the PetCO_2_ and MRO_2_ measured at the same points as Table 3. Note that PetCO_2_ actually decreased at the end of recovery so cannot explain this ventilation increase. Similarly, MRO_2_ change was small and could not account for the ventilation increase. Table 3 is similar to Table 2, but is a paired comparison for the same exercise level breathing air. A mean change of 4.3 L/min was measured which was about half the effect of combined exercise and 3% CO_2_ inhalation. These results will be discussed later.

**TABLE 2:** Paired comparison of changes in ventilation, PetCO_2_, and MRO_2_‐45% max exercise with 3% CO_2_ inhalation

Subject	Ventilation (L/min)		PetCO_2_ (mmHg)		MRO_2_ (ml/min)	
	Pre	Post	Pre	Post	Pre	Post
1	31.6	47.9	51.5	45.7	984	973
2	36	37.5	46.7	47.4	869	880
3	42.2	48.4	48.9	45.2	940	1008
4	38.6	45.8	47.7	46.8	991	977
5	48.8	55.2	47.6	45.8	1066	1103
6	35.2	48.6	49.4	45.1	952	1011
7	41.2	49.3	48.4	48	980	1010
Mean change		8.44		−2.31		25.7
SD		4.92		2.35		32.1
*t*(6)		4.5		2.6		2.12
*p*		<0.01		<0.05		

**TABLE 3:** Paired comparison of baseline changes in ventilation, PetCO_2_, and MRO_2_‐45% Max exercise breathing air

Subject	Ventilation (L/min)		PetCO_2_ (mmHg)		MRO_2_ (ml/min)	
	Pre	Post	Pre	Post	Pre	Post
1	28.9	35.6	43.4	40.4	1013	1158
2	26.8	32.2	42.7	41.2	923	1032
3	29.9	37.9	41.1	38.3	880	1052
4	28.4	30.5	42.6	42.2	1006	977
5	38.1	37.5	40.2	39.5	1070	1111
6	28.1	33.8	41.8	40.3	964	1103
7	31.4	34.4	41.4	40.4	951	1031
Mean change		4.33		−1.56		93.9
SD		2.99		1.01		69.6
*t*(6)		3.83		4.1		3.57
*p*		<0.01		<0.01		<0.02

## 4 DISCUSSION

The use of rebreathing during exercise to test CO_2_ responses has been popular due to the simplicity of administration. However, interpretation of the divergent results has been difficult. The first factor to consider is inspired CO_2_ level. The experimental protocol followed by Miyamura et al. (1976) will be used as an example. Resting rebreathing started with an initial CO_2_ percentage of 7% as compared to 9%–10% for exercise. These inspired levels especially for exercise are in the range where a negative reaction can result (Miyamura et al., 1976). This could explain depression effects. The time duration of rebreathing is the next factor. Four min was used for rest and 1.5–2 min for exercise. The average time constant for the central chemoreceptors is about 3 min (Bellville et al., 1979). This means that resting responses could be closer to steady state and larger than exercise just due to differences in allowed equilibration times. The ratio (1−exp (−1.5/3))/(1−exp (−4/3)) = 0.53 is the predicted step response difference just due to the different 1.5 and 4 min equilibration times. So central chemoreceptor exercise rebreathing responses would be underestimated by about 50% in a comparison. Next to consider is the range of PetCO_2_ covered. The resting range was 50–70 mmHg as compared to 60–95 mmHg for exercise. The non‐linear shapes of several of the exercise responses were clearly visible in the published plot. At high CO_2_ levels saturation is apparent and can easily decrease sensitivity by another 50%. A comparison of response slopes is then questionable in significance.

Duffin et al. (1980) have questioned the validity of using PetCO_2_ as an index of stimulating central chemoreceptors during rebreathing and concluded that exercise does not increase chemosensitivity. Rebreathing was not used in the present study so there was a difference in methodology. Our inhaled CO_2_ was limited to 3% while the starting rebreathing level used by Duffin et al. (1980) was 7% who noted that three of their subjects could not complete the exercise rebreathing test due to reaching maximum ventilation levels. Maximum VE was not reached in our subjects despite similar exercise level (40 instead of 50 watts). Also, our conclusions do not rely on CO_2_ inhalation effects during exercise since an enhanced CO_2_ sensitivity was found during exercise with air breathing. The only comparison made was to 3% CO_2_ inhalation at rest. To better estimate PaCO_2_ we used the empirical formula of Jones based on measured end‐tidal and tidal volume which was validated in normal exercising subjects as leading to agreement to within 1.04 mmHg of direct samples of arterial blood.

The current study compared 3% CO_2_ inhalation in a sequential manner from rest, 40W, 45% VO_2 max_ all with durations of 5–6 min. A similar exercise sequence was also used with the subjects breathing air. All seven subjects tolerated the protocol without complaint. The upper range of PetCO_2_ was below 55 mmHg. Non‐linear effects related to CO_2_ was not observed for this rest moderate exercise sequence.

The current results agreed with the previous conclusion (Weil et al., 1972) that chemosensitivity was enhanced during mild exercise and was not significantly increased at higher levels of exercise. VE during the on transient of mild exercise also closely correlated with VCO_2_ rate dynamically. This correlation in time has been previously discussed (Whipp, 2007), but not specifically used to predict VE as in Figure 2. The more recent results of Poon and Greene (1985) added controlled PetCO_2_ during exercise and also confirmed enhanced chemosensitivity during exercise.

**FIGURE 5:**
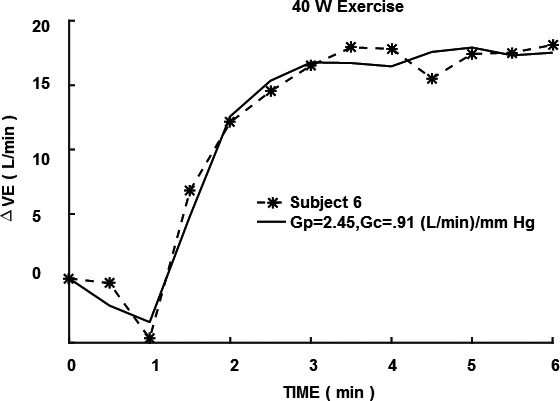
Individual subject 40W exercise response fitted with model. VE, minute ventilation; *G*_p_, peripheral gain; *G*_c_, central gain

To test how chemosensitivity enhancement could affect exercise responses, a previous model of central and peripheral dynamics (Bellville et al., 1979) was fitted to mild exercise data during air breathing (0% CO_2_ inhalation). The measured PetCO_2_ values were used as input and VE responses were used as output to estimate *G*_c_ and *G*_p_ according to a least squares model fit. A close fit was obtained as shown in Figure 3. The total chemoreceptor gains estimated were about a factor of two higher than the measured resting gain (Figure 1). The close fit to VE of Figure 3 and correlation of VE to VCO_2_ rate (Figure 2) implied that increased chemoreceptor gains could be the underlying mechanism behind this matching. Individual subject exercise responses could also be closely fitted as shown in Figure 5. A complete summary of the individual fits is shown in Table 4. Two parameters *G*_p_ and *G*_c_ were adjusted for a least squares fit as indicated for Figure 3. The sum of the two was compared to the resting sensitivity (Grest) as listed in Table 1 for each subject. The paired comparison showed a mean increase from rest of 1.79 (L/min)/mmHg which was a statistically significant change (*p *< 0.01). The *G*_p_ during exercise was consistently larger than Grest for all subjects with a mean paired difference of 1.04 (L/min)/mmHg (*p *< 0.01). The results were consistent with a significant increase in peripheral chemoreceptor sensitivity in method (during mild exercise). Central chemosensitivity must account for the mean difference of 1.79−1.04 = 0.75 (L/min)/mmHg. In conclusion, both peripheral and central chemosensitivity to CO_2_ increased in mild exercise with the peripheral chemoreceptors playing a dominant role. This was a previously unreported result that demonstrated the utility of the dynamic model fitting method (Bellville et al., 1979). Use of 3% inhaled CO_2_ during mild exercise does not lead to secondary non‐linear effects or require different equilibration times for rest and exercise.

**TABLE 4:** Exercise subject chemosensitivity ([L/min]/mmHg)

Subject	*G*_rest_	*G*_p_	*G*_c_	*G*_p_ + *G*_c_	(*G*_p_ + *G*_c_)−*G*_rest_	*G*_p_−*G*_rest_
1	0.12	2.09	0.42	2.51	2.39	1.97
2	1.95	2.33	0.65	2.98	1.03	0.38
3	1.54	1.99	1.21	3.20	1.66	0.45
4	0.61	1.56	0.77	2.33	1.72	0.95
5	0.71	1.63	1.21	2.84	2.13	0.92
6	1.70	2.45	0.91	3.36	1.66	0.75
7	0.90	2.73	0.10	2.83	1.93	1.83
Mean	1.08	2.11^§^	0.75	2.86*	1.79	1.04
SD	0.67	0.43	0.41	0.36	0.43	0.63

*G*_rest_, resting gain; *G*_p_, peripheral gain; *G*_c_, central gain.

* *p* < 0.001, *G*_p_ + *G*_c_ versus *G*_rest_. *t*_(6)_ = −10.995, *p* = 0.000, *d* = 3.310.

§ *p* < 0.01, *G*_p_ versus *G*_rest_. *t*_(6)_ = −4.352, *p *= 0.005, *d* = 1.830.

Long‐term facilitation of ventilation has attracted considerable attention due to the likelihood of leading to brain serotonin release (Mihorn et al., 1980). Chemoreceptor stimulation in animals was the initial focus and repetitive stimulations were found necessary to produce measurable facilitation which was long lasting. In humans, intermittent hypoxia with a background of hypercapnia was found to be effective to produce LTF (Griffin et al., 2012; Mitchell & Johnson, 2003; Wadhwa et al., 2008). Since the current and previous reports showed enhanced chemoreceptor stimulation in exercise, it seemed possible that adding hypercapnia might lead to LTF. The current results were consistent with LTF when the baseline involved light exercise (40W) and a step change was made to 45% maximum MRO_2_ for 6 min followed by a step return to 40 W exercise while inhaling 3% CO_2_. This on followed by off can then be classified as an intermittent stimulation except it is not repeated. An average ventilation facilitation of 8.4 L/min was measured in seven subjects with no significant change in MRO_2_ and a decrease in PetCO_2_ of 2.3 mmHg. The decrease in PetCO_2_ was interpreted as indicating a larger facilitation was predicted by correcting for CO_2_ sensitivity which was measured (Table 4‐combined *G*_c_+*G*_p_). Thus, up to a predicted 8.4 + 2.3 × 2.9 = 15.1 L/min of LTF can be justified. Something other than PetCO_2_ or MRO_2_ was then responsible for this ventilation change. LTF is a possible explanation. The "off" ventilation response to moderate exercise was remarkable in that the immediate response fell below the control level before increasing. This was observed with or without 3% CO_2_ inhalation. In exercise this immediate response can be tied to a neurally mediated rate sensitivity (Yamashiro & Kato, 2014). The ensuing increase following the immediate decrease may be due to a transition not to rest but light exercise. The "on" ventilation transient does not show such a large initial transition, so rate sensitivity was only present during the "off" transient. Transition to rest following an exercise usually shows a small rapid neurally mediated decrease followed by a slower decrease back to resting ventilation. The transient change between light and moderate exercise and inhalation of 3% CO_2_ appears to involve a strong neural rate sensitivity component tied to exercise which is absent from prior intermittent hypoxia trials. This may be relevant because LTF is a neurally mediated response.

Part of the neural response to exercise which is completely different from hypoxic and hypercapnic responses is the effect on functional residual capacity (FRC) (Cha et al., 1987). The connection to ventilation is because the FRC decrease occurring in light exercise (Cha et al., 1987) is tied to an increased tidal volume of 7% vital capacity (approximately 350 ml for a human subject) which can account for a ventilation of 8.8 L/min for a breathing frequency of 25 breaths/min expected in light exercise. Such increased ventilation and decreased FRC requires involvement of the expiratory muscles. This ventilation change is close to what was measured following recovery to moderate exercise and 3% CO_2_ inhalation. Both hypoxia and hypercapnia have been reported to increase FRC by 14%–15% (Garfinkel & Fitzgerald, 1978). Thus, during combined CO_2_ inhalation and moderate exercise FRC effects can cancel out. When moderate exercise ends and light exercise resumes the FRC decrease effect can dominate explaining increased ventilation. While CO_2_ inhalation as used in the present study does continue even in light exercise, the effect of CO_2_ inhalation is not additive and is significantly larger due to the higher exercise ventilation level in moderate exercise.

Exercise of 45% Maximum while breathing air resulted in a ventilation increase of 4.3 L/min (*p *< 1%) and a decrease in PetCO_2_ of 1.6 mmHg (*p *< 1%). Again, the increase in ventilation cannot be accounted for by PetCO_2_. MRO_2_ did increase by 93.9 ml/min (*p *< 2%), but based on the measured respiratory quotient of 0.9 and measured correlation of ventilation and MRCO_2_ as shown in Figure 2 (0.02663), the estimated ventilation increase was 0.9 × 93.9 × 0.02663 = 2.3 L/min. Thus, exercise alone after accounting for MRCO_2_ change was 4.3 – 2.3 = 2 L/min. Accounting for the decrease in PetCO_2_ of 1.6 mmHg will increase the estimate to 2 + 1.6 × 2.9 = 6.6 L/min. This is 44% of the 15.1 L/min estimated for combined exercise and 3% CO_2_ inhalation. We conclude that combined 45% maximum exercise and 3% inhaled CO_2_ for 6 min followed by a return to light exercise was effective in stimulating augmented ventilation resembling LTF. This conclusion is also consistent with the previous report of enhanced LTF when a background of hypercapnia is used during chemoreceptor stimulation by intermittent hypoxia (Harris et al., 2006).

Facilitation of ventilation following passive exercise has been reported in a case study of a paralyzed human subject (Nash et al., 2004). Treadmill exercise was applied for 7 min duration with robotic assistance. Reported ventilation was 7.2 before, 9.6 during, and 9.2 L/min following with measured oxygen consumption returning to control levels immediately following exercise. This report is consistent with the current findings. Mitchell and Johnson (2003) have reviewed respiratory neural plasticity and cited some animal studies supporting such plasticity in hypercapnic exercise, but experimental evidence in man was lacking.

The chemosensitivity and LTF responses during hypercapnic exercise appears highly sensitive to levels of exercise, hypercapnia, and durations of stimulation. This is based on the relative paucity of positive reports. Responses can then range from depression to enhancement. Three percent inhaled CO_2_ appears to be a good choice to produce enhancement. Also, the baseline exercise of 40 W and step level of 45% max MRO_2_ for 6 min appears effective as well for enhancement of both effects.

One limitation of the current results was only male subjects were used, so the effect of subject sex is unknown. The studied subjects appeared to be all healthy and not on any medication which might influence the results.

## CONFLICT OF INTEREST

No conflicts of interest, financial or otherwise, are declared by the author (s).

## AUTHOR CONTRIBUTIONS

S.Y. and T.K. conception and model development. T.K. and T.M. was responsible for collecting the experimental data. S.Y. drafted manuscript. T.K. edited and revised manuscript. S.Y., T.K., and T.M. approved final version of manuscript.

## APPENDIX A

Central and peripheral chemoreceptor model

The model used was a simplified version of Bellville et al. (1979). It consisted of two differential equations:

Variables: *y*_p_ = ventilation change due to peripheral chemoreceptor = DVEp; *y*_c_ = ventilation change due to central chemoreceptor = DVEc; DVE = *y*_p_ + *y*_c_ = change in total ventilation from resting level in L/min; DPetCO_2_ = change in PetCO_2_ from resting level in mmHg; *G*_p_ = peripheral gain in (L/min)/mmHg; *G*_c_ = central gain in (L/min)/mmHg; *T*_p_ = peripheral time constant in min; *T*_c_ = central time constant in min; *T*d_p_ = peripheral time delay in min; *T*d_c_ = central time delay in min; xin = DPetCO_2_.

(A1) $$T_{p}\;dy_{p}/dt=\text{xin}\;\left(t‐T_{\text{dp}}\right)*G_{p}‐y_{p},$$

(A2) $$T_{c}\;dy_{c}/dt=\text{xin}\;\left(t‐T_{\text{dc}}\right)*G_{c}‐y_{c}.$$

Both of the above equations are first order and can be simulated in Matlab using transfer functions defined as:

$$H_{p}=\text{tf}\left(\left[0\;G_{p}\right],\left[T_{p}\;1\right],\text{‘Input}\;\text{Delay’},Td_{p}\right);\;\text{for A}1,$$

$$H_{c}=\text{tf}\left(\left[0\;G_{c}\right],\left[T_{c}\;1\right],\text{‘Input}\;\text{Delay’},Td_{c}\right);\;\text{for A}2.$$

Simulation outputs were obtained by:

$$\left[y_{p},∼\right]=\text{lsim}\left(H_{p},\text{xin},t,0\right);$$

$$\left[y_{c},∼\right]=\text{lsim}\left(H_{c},\text{xin},t,0\right);$$

where *t* = solution time in minutes and 0 sets the initial condition to 0.

All of the temporal parameters were set to normal values as given by Bellville et al. (1979) and kept constant throughout. *G*_p_ and *G*_c_ were adjusted to minimize the least squares difference between measured and model predicted ventilations. The Matlab function fminsearch was used in combination with the above transfer function models for least square fitting. The fixed temporal constants were:

$$T_{p}=0.15,Tc=1.67,Tdp=0.2,\;\text{and}\;Td_{c}=0.2\;\text{all in minutes}.$$
